# Lymphatic Drainage-Promoting Effects by Engraftment of Artificial Lymphatic Vascular Tissue Based on Human Adipose Tissue-Derived Mesenchymal Stromal Cells in Mice

**DOI:** 10.1155/2023/7626767

**Published:** 2023-11-06

**Authors:** Yoshiya Asano, Hiroshi Shimoda, Daisuke Okano, Michiya Matsusaki, Mitsuru Akashi

**Affiliations:** ^1^Department of Neuroanatomy, Cell Biology and Histology, Hirosaki University Graduate School of Medicine, 5 Zaifu-cho, Hirosaki, Aomori 036-8562, Japan; ^2^Department of Anatomical Science, Hirosaki University Graduate School of Medicine, 5 Zaifu-cho, Hirosaki, Aomori 036-8562, Japan; ^3^Department of Applied Chemistry, Graduate School of Engineering, Osaka University, 2-1 Yamada-oka, Suita, Osaka 565-0871, Japan; ^4^Building Block Science, Graduate School of Frontier Biosciences, Osaka University, 1-3 Yamada-oka, Suita, Osaka 565-0871, Japan

## Abstract

Regenerative medicine using lymphatic vascular engineering is a promising approach for treating lymphedema. However, its development lags behind that of artificial blood vascular tissue for ischemic diseases. In this study, we constructed artificial 3D lymphatic vascular tissue, termed ASCLT, by co-cultivation of ECM-nanofilm-coated human adipose tissue-derived mesenchymal stromal cells (hASCs) and human dermal lymphatic endothelial cells (HDLECs). The effect of hASCs in lymphatic vessel network formation was evaluated by comparison with the tissue based on fibroblasts, termed FbLT. Our results showed that the density of lymphatic vascular network in ASCLT was higher than that in FbLT, demonstrating a promoting effect of hASCs on lymphatic vascular formation. This result was also supported by higher levels of lymphangiogenesis-promoting factors, such as bFGF, HGF, and VEGF-A in ASCLT than in FbLT. To evaluate the therapeutic effects, FbLTs and ASCLTs were subcutaneously transplanted to mouse hindlimb lymphatic drainage interruption models by removal of popliteal and subiliac lymph nodes. Despite the restricted engraftment of lymphatic vessels, ASCLT promoted regeneration of irregular and diverse lymphatic drainage in the skin, as visualized by indocyanine green imaging. Moreover, transplantation of ASCLT to the popliteal lymph node resection area also resulted in lymphatic drainage regeneration. Histological analysis of the generated drainage visualized by FITC-dextran injection revealed that the drainage was localized in the subcutaneous area shallower than the dermal muscle. These findings demonstrate that ASCLT promotes lymphatic drainage in vivo and that hASCs can serve as an autologous source for treatment of secondary lymphedema by tissue engineering.

## 1. Introduction

Secondary lymphedema is caused by surgical lymphadenectomy and/or radiation in cancer therapy. This results in poor quality of life due to failure of peripheral lymphatic drainage, followed by severe swelling and limitation of movement. Progression of symptoms often leads to elephantiasis with fibrosis of the skin or concurrence of cellulitis, which increases the risk of life-threatening infections [1–3]. Secondary lymphedema has traditionally been treated by symptomatic therapies such as massage or use of pressure supporters for lymphatic drainage [4]. Since the 1990s, lymphaticovenular anastomosis using microsurgery technique has been developed. This therapeutic method is effective but cannot be used for advanced stages of edema [5]. Recently, vascularized lymph node transfer, a regenerative medicine using self-derived organs, has also been applied in cases of aggravated symptoms. However, this method requires lymph node excision. It is highly invasive and sometimes ineffective [6].

Regenerative medicine by administration of multipotent mesenchymal stromal cells or engineered lymphatic vascular tissues is a novel option for lymphedema treatment, but it has not yet reached clinical application [7, 8]. Regarding this technique, several attempts have been made to inject mesenchymal stromal cells for stimulating lymphangiogenesis by their secretory factors or to transplant artificial lymphatic vascular tissue [7, 9, 10]. In the latter case, the engineered lymphatic vascular tissues have been constructed based on the scaffolds including hydrogel, fibrin gel, and collagen [11–13]. However, compared with the artificial blood vascular tissue for ischemic diseases, the development of lymphatic vascular engineering lags behind [7].

Regarding the tissue engineering for regenerative medicine of lymphatic drainage, we have reported on the engraftment of artificial lymphatic vascular tissue constructed by cell accumulation technique, which is based on cellular coating by matrix proteins, termed ECM-nanofilm coating [14–16]. In these reports, the artificial three-dimensional (3D) tissues were constructed using only coated culture cells, without the addition of any scaffolds or angiogenetic factors other than fetal bovine serum. By this method, the vascular networks are formed in at least four days [16]. These are thought to be achieved in a microenvironment of 3D cultured cells where the expression of angiogenic factors is promoted [14]. The lymphatic vascular tissue developed by our group was constructed by co-cultivation of normal human dermal fibroblasts (NHDFs) and human dermal lymphatic endothelial cells (HDLECs) with ECM-nanocoating [14, 17]. The constructed vessel networks have a lymphatic capillary-like tubular structure, and the tissue can be engrafted under the skin or on the fascia of nude mice [17]. These results suggest that our constructed tissue has therapeutic potential in regenerative medicine for intractable lymphedema.

In the clinical application of human 3D artificial tissue transplantation, it is necessary to avoid rejection and to provide safe cells that are free from cancerous transformation. For this purpose, the use of autologous cells would lead to an ideal regenerative medicine [18]. Human adipose tissue-derived mesenchymal stromal cells (hASCs) are multipotent cells that can be harvested with minimal invasive procedures such as liposuction from subcutaneous fat. They have been applied in regenerative medicine through cell transplantation due to their secretion of regeneration-promotive factors, potential for differentiation into multiple structures, and immunomodulatory activities [19, 20]. In particular, blood and lymphatic vessel regenerative activities of hASC have been reported from cell transplantation [10, 15, 21, 22]. In our previous study, we have constructed artificial blood vascular tissue by using hASCs and human umbilical vein-derived endothelial cells (HUVECs), and successful engraftment of this tissue in nude mice was confirmed [16]. Therefore, the efficacy of hASCs for the construction and engraftment of artificial lymphatic network is also expected.

In this study, we constructed artificial 3D lymphatic vascular tissue (termed ASCLT) by co-culturing hASCs and HDLECs coated with ECM-nanofilm as a regenerative medicine for lymphatic drainage disorders. The effect of hASCs on lymphatic vessel network formation was evaluated by comparison with NHDF-based tissue (termed FbLT). Then, the effect of tissue transplantation on lymphatic drainage regeneration was investigated by using a mouse lymph node resection model.

## 2. Materials and Methods

### 2.1. Cell Culture

Primary cultured human cells were used in this study. Human mesenchymal stem cells from adipose tissue were purchased from PromoCell (Heidelberg, Germany). These cells were used as hASCs referred to as human adipose tissue-derived mesenchymal stromal cells in this study. Neonatal NHDFs and HDLECs were purchased from Lonza (Walkersville, MD). Dulbecco's modified Eagle medium (DMEM, Wako Pure Chemical, Osaka, Japan) containing 10% fetal bovine serum (FBS, SAFC Biosciences; Lenexa, Kansas) was used to culture NHDFs. Endothelial Growth Medium-2 (Lonza) and Mesenchymal Stem Cell Growth Medium 2 (Takara Bio Inc., Kusatsu, Japan) were used to culture HDLECs and hASCs, respectively. CultureSure Freezing Medium was purchased from FUJIFILM Wako Pure Chemical (cat. no. 039-23511, Osaka, Japan). Transwell inserts for 24-well culture plate (6.5 mm of diameter) with a porous polyester bottom (pore size: 0.4 *μ*m) (cat. no. 3470, CORNING Inc., New York, NY) were used for the construction and cultivation of 3D artificial human tissues. Cells were cultured or maintained at 37°C and 5% CO_2_.

### 2.2. Reagents

Bovine plasma-derived fibronectin (FN) and porcine skin gelatin (G) were purchased from FUJIFILM Wako Pure Chemical Corporation, Ltd. All antibodies used in this study are listed in Supporting Information 1. Indocyanine green (ICG; Diagnogreen for injection) and dextran (2000 kDa)-conjugated fluorescein (FITC-dextran, cat. no. D7137) were purchased from Daiichi Sankyo (Tokyo, Japan) and Thermo Fisher Scientific (Waltham, MA), respectively.

### 2.3. Animals

Nude mice (BALB/cAJcl-nu, female at 6 weeks of age) were purchased from CLEA Japan, Inc. (Tokyo, Japan). Low-autofluorescence diet iVid#2 was purchased from Oriental Yeast (Tokyo, Japan). Mice were maintained under controlled conditions at 12 h light:dark cycle and at 21°C temperature. All animal experiments in this study were approved by the Animal Research Committee at Hirosaki University and were conducted according to the Guidelines for Animal Experimentation, Hirosaki University.

### 2.4. Construction of 3D Artificial Human Lymphatic Vascular Tissue

The artificial lymphatic vascular tissues were constructed using a cryopreserved cell accumulation method (CP-CAM), based on layer-by-layer cell coating technique [14, 16]. In brief, cultured hASCs and NHDFs (within 8 passages each) were collected, coated layer-by-layer with fibronectin and gelatin (ECM nanofilm) on each cell, and then cryopreserved in CultureSure Freezing Medium for further use [16]. On the other hand, cultured HDLECs were collected, coated with ECM nanofilm, and immediately used for tissue construction. For construction of ASCLT, coated and cryopreserved hASCs were thawed and seeded in three dimensions with coated HDLECs in the “sandwich” or “mixture” manner. In the sandwich manner, one layer of HDLECs (1 × 10^5^ cells) was laminated between the upper and lower four layers of hASCs (1 × 10^5^ cells/layer) in a transwell insert. In the mixture manner, 8 × 10^5^ of hASCs and 1 × 10^5^ HDLECs were mixed and seeded in a transwell insert. For the construction of FbLT, NHDFs were used instead of hASCs by the same protocols as described above. The seeded cells were cultured in DMEM containing 10% FBS at 37°C with 5% CO_2_ for four days to allow lymphatic network formation.

### 2.5. Analysis of Lymphatic Vascular Network Structure in Artificial Tissues

The ASCLTs and FbLTs were prepared in a “sandwich manner” as described in Section 2.4. For fluorescent microscopy, the constructed artificial tissue was cut from transwell insert and fixed in 4% paraformaldehyde in 0.1 M phosphate buffer (pH 7.4). Tissue sections were then prepared, and immunostaining for human CD31, Prox-1, and human podoplanin was performed according to the previously reported methods [17]. The samples were observed using a fluorescence microscope BZ-X700 (Keyence, Tokyo, Japan). Quantitative analysis of total vascular length, number of junctions, and vessel area in lymphatic vascular network was performed using ImageJ software (https://imagej.nih.gov/ij/download.html) and Angiogenesis Analyzer (https://imagej.nih.gov/ij/macros/toolsets/Angiogenesis%20Analyzer.txt), a plugin program for ImageJ [23]. The samples were also observed using a confocal microscope Nikon C2 (Nikon, Tokyo, Japan). Analysis of the 3D network was performed by using Fiji software (https://imagej.net/Fiji).

For transmission electron microscopy, the artificial tissues were fixed in 2.5% glutaraldehyde and 2% paraformaldehyde in 0.1 M phosphate buffer (pH 7.4) at 4°C and embedded in Epon 812 (Nisshin EM, Tokyo, Japan) as previously described [16, 24]. The transverse sections for light microscopy were prepared at 500 nm thickness and stained by 0.5% toluidine blue. Ultra-thin sections for electron microscopy were prepared at 70 nm thickness using an ultramicrotome (Reichert Ultracut S, Leica, Wetzlar, Germany) and stained with 4% uranyl acetate and lead stain solution (Sigma-Aldrich, St. Louis, MO). The samples were then observed under a transmission electron microscope (JEM-1230, JEOL, Tokyo, Japan).

### 2.6. Analysis of Angiogenesis-Related Factor Profiles

The ASCLTs and FbLTs in the “sandwich manner” were cultured in 2.3 ml of DMEM for 24 hours and the culture supernatants were collected. Moreover, the culture supernatants of artificial tissue constructed by overlaying 8 layers of hASCs or NHDFs alone (termed ASCTs or FbTs, respectively) were also examined for the data without HDLECs. The expression of 55 angiogenesis-related factors in culture supernatants of artificial tissues was analyzed by dot-blot assay using Proteome Profiler Human Angiogenesis Array Kit (R&D Systems, Minneapolis, MN) according to the manufacturer's protocol. The detected intensity of each factor was quantified by Fiji software (https://imagej.net/Fiji). For each factor, the blots were performed in duplicate and the average values are presented.

### 2.7. Preparation of Mouse Lymphatic Drainage Interruption (LDI) Model

Mouse hindlimb-gluteal LDI model was prepared by resecting the popliteal and ipsilateral subiliac lymph nodes of BALB/c nude mice. The schemas for lymph nodes and LDI model preparation are shown in Supporting Information 2. For LDI model preparation, mice were anesthetized by intraperitoneal administration with medetomidine hydrochloride (0.3 mg/kg), midazolam (4 mg/kg), and butorphanol tartrate (5 mg/kg), and then 10 *μ*l of 2% patent blue in phosphate buffered saline (PBS) was injected into the left plantar to visualize the left popliteal lymph node. Surgical resection of the popliteal lymph node was performed, resulting in a deficiency of lymphatic drainage from left hindlimb to the gluteal. In addition, ipsilateral subiliac lymph node was dissected to reduce the random lymphatic drainage toward this lymph node, as evaluated from preliminary experiments (data not shown). Mice were maintained for at least 1 week to allow the injured skin to recover and were fed with low-autofluorescence diet, iVid#2. For in vivo lymphatic drainage imaging, 10 *μ*l of ICG solution (0.5 mg/ml in PBS) was injected into the plantar and instep of foot. At 30 minutes after the injection, the mice were anesthetized, and then the lymphatic drainage was observed by in vivo macro imaging system Lumazone (Nippon Loper, Tokyo, Japan) using 810 nm and 890 nm for excitation and emission wavelength, respectively. The intact lymphatic drainage in intact mice was shown by detection of ICG at popliteal lymph nodes, external sacral lymph nodes (at the gluteal), and the drainage between them (Supporting Information 2b). The resection of the lymph nodes resulted in the disappearance of ICG drainage reaching the external sacral lymph node (Supporting Information 2c). ICG-positive drainage appearing after lymph node resection was considered as the regenerated lymphatic drainage.

### 2.8. Transplantation of ASCLT to Mouse LDI Model

The ASCLTs were prepared in the “mixture manner” as described in Section 2.4 and transplanted to mouse LDI model. In the present study, two methods of transplantation were examined. First, the artificial tissue was cut from the transwell insert with porous polyester bottom and subcutaneously transplanted into the left gluteal where the lymphatic drainage was disrupted (gluteal transplantation, Supporting Information 2d). Second, the artificial tissue was cut from the transwell insert with removal of the polyester bottom and transplanted into deep popliteal tissue where the popliteal lymph node was dissected (popliteal transplantation, Supporting Information 2e). The transplantation was performed while mice were under anesthesia as described above. For immunosuppression, cyclosporin A (Neoral, Novartis, Rueil-Malmaison, France) was added to the drinking water (with a final concentration of 120 mg/L) one week before and throughout the engraftment period as previously reported [16]. After the transplantation, the in vivo lymphatic drainage imaging by ICG injection was performed as described in Section 2.7.

### 2.9. Analysis of ASCLT-Transplanted Tissues and Lymphatic Drainage

The gluteal dermal tissues or hindlimbs with transplanted ASCLT were fixed in 4% paraformaldehyde in 0.1 M phosphate buffer (pH 7.4), embedded in paraffin, and then sectioned at 5 *μ*m thickness. The hindlimbs were decalcified by 10% EDTA-Na prior to embedding. The immunohistochemistry was performed according to the methods in our previous study [17].

Lymphatic drainage was histologically analyzed using FITC-dextran according to the previous study [25]. After four weeks of popliteal ASCLT transplantation, 10 *μ*l of FITC-dextran was subcutaneously injected to the plantar of the hindlimb. At 30 minutes of injection, the mice were sacrificed, and the legs and the abdominal skin where the lymphatic drainage was observed by ICG imaging were collected. The lymphatic drainage in abdominal skin was observed from subcutaneous side using fluorescence stereomicroscope MVX10 (Olympus, Tokyo, Japan) with excitation of FITC. Then the leg and skin were embedded in paraffin, sectioned, and immunostained for FITC.

### 2.10. Statistical Analysis

The statistical analysis between two groups of data was performed by two-tailed *t*-test. The number of samples in each analysis was shown in the figure legends. The normal distribution in all sample data was confirmed by the degree of skewness and kurtosis, and the equality of variance in the two groups of data was validated by F-test. Student's *t*-test or Welch's *t*-test was used, depending on the data with equal variances or unequal variances, respectively. The statistical analysis for more than two groups of the data was performed by one-way ANOVA followed by Bonferroni post hoc test. *P* values of less than 0.05 are considered statistically significant.

## 3. Results

### 3.1. Lymphatic Vascular Structure in ASCLT

**Figure 1:**
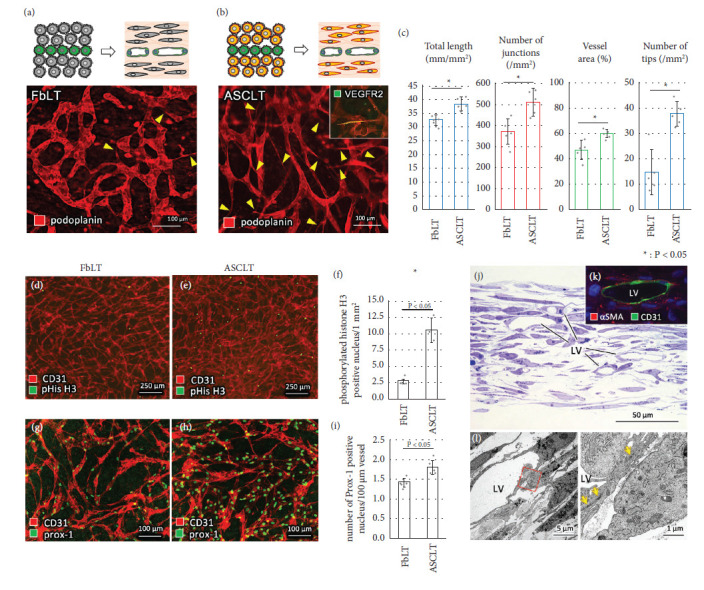
Lymphatic vascular structure in FbLT and ASCLT. FbLT (a) and ASCLT (b) were constructed by the sandwich manner (green: HDLECs; grey: fibroblasts; orange: hASCs), and the lymphatic vascular structure was compared. The artificial lymphatic networks are visualized by immunostaining for podoplanin. The arrow heads indicate the formation of tip structure suggesting the sprouting of the vessels. VEGFR2 is detected in the tip of ASCLT ((b), inset). (c) Quantitative analysis of lymphatic vascular structures (*n* = 5). The ASCLT shows higher score in total length, number of junctions, vessel area, and number of tips than FbLT. (d–f) The frequency in phosphorylated histone H3- (pHis H3-) positive cells (*n* = 5). The lymphatic vascular networks visualized by immunostaining for CD31. (g–i) The density of endothelial cells in the lymphatic vascular networks visualized by immunostaining for prox-1 and CD31 (*n* = 5). (j) Epon-embedded ASCLT was sectioned and stained with toluidine blue. The tubular structures (LV) are observed. (k) Section of paraffin-embedded ASCLT was immunostained for *α*SMA and CD31. No *α*SMA-positive cells surrounding CD31-positive vessels are observed. (l) TEM image of ASCLT. The closed tubular structures (LV) are formed by interendothelial junctions (arrows).

For in vitro structural analysis, the artificial lymphatic vascular tissues were constructed by the “sandwich manner” in which one layer of HDLECs was co-cultured between the four upper and lower layers of NHDFs or hASCs (Figures 1(a) and 1(b)). The artificial tissues were fixed on day 4 after cell seeding, and their vascular network was visualized by immunostaining for human podoplanin. In FbLT, the formation of the vessel network-like lymphatic capillaries was observed corresponding to our previous studies [17, 24]. In ASCLT, a podoplanin-positive vessel network was also found demonstrating the formation of lymphatic vessels. However, morphological differences in the vessel network were found between FbLT and ASCLT. In particular, tip structure was remarkably found in ASCLT compared to FbLT (Figures 1(a) and 1(b), arrowheads). The immunostaining for VEGFR2 was sporadically found in the tip structures of ASCLT (Figure 1(b), inset). The immunostaining for VEGFR3 in ASCLT was also performed, but only faint staining was observed throughout the vessels (data not shown). Quantitative analysis showed that the vessel network in ASCLTs had higher total length, number of junctions, vessel area, and number of tip structures than FbLTs (Figure 1(c)).

In relation to the significantly high density of vessel network in ASCLT, immunostaining for phosphorylated histone H3 was observed more frequently in ASCLTs than FbLTs (Figures 1(d)–1(f)). Correspondingly, the density of prox-1-positive nuclei in the vessel network was higher in ASCLTs than in FbLTs (Figures 1(g)–1(i)).

The transverse sections of ASCLT revealed the distribution of tubular vessel structures (Figure 1(j)). Immunostaining showed no *α*SMA-positive cells surrounding CD31-positive vessel structures (Figure 1(k)). Electron microscopy showed that lymphatic endothelial cells formed closed tubular structures by interendothelial adherens junctions (Figure 1(l)).

### 3.2. The Effect of hASCs in Promoting Artificial Lymphatic Vascular Network Formation

**Figure 2:**
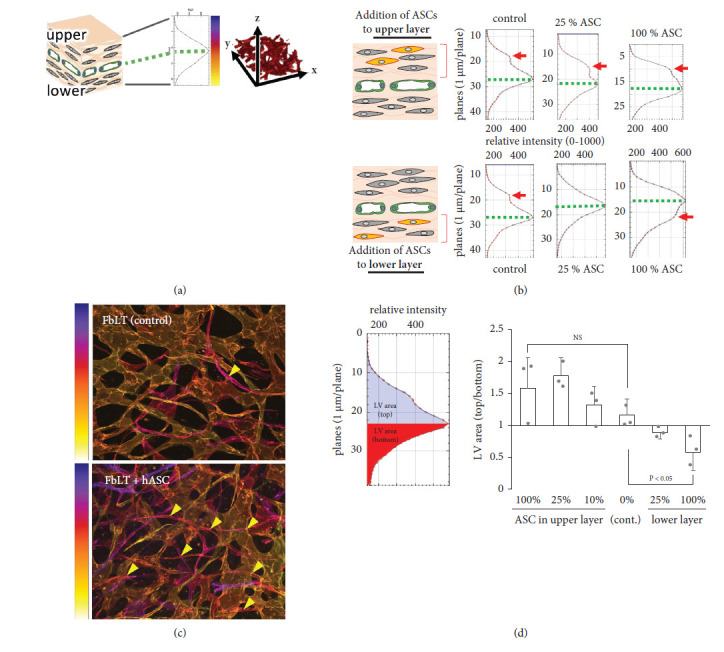
The effects of hASCs on artificial lymphatic vascular network formation. (a, b) The lymphatic vascular formation-promoting effect of hASCs was evaluated by addition to upper or lower layer of FbLT (green: HDLECs; grey: fibroblasts; orange: hASCs). The lymphatic vascular structure was immunostained for CD31, and then the density of the vascular structure along *Z* axis was quantified as shown in the graphs (b). Green dotted lines indicate the main peak of vascular structure upon HDLEC-seeded level. Arrows indicate the extra peaks of the vascular structure. (c) The lymphatic vascular networks of FbLT with and without an addition of 25% hASCs in the upper layer. The networks were immunostained for CD31, and then the confocal image was analyzed using Fiji software; the *Z* axis level of network structures was colored according to the left color bar. Arrow heads indicate the sprouting of lymphatic vessels in the upper layer. Addition of 25% hASCs resulted in an increase of the sprouting. (d) Quantitative analysis of increased lymphatic vascular structures by addition of hASCs at several amounts (*n* = 3). ANOVA with Bonferroni post hoc test showed significant difference of lymphatic vascular density by addition of hASCs to the lower layer (0%, 25%, and 100%, *P* < 0.05). By addition of hASCs to the top layer (0%, 10%, 25%, and 100%), tendency of difference was shown, although it was not significant (NS).

To analyze the effect of hASCs in promoting lymphatic vascular network formation, various concentrations of hASCs were added into the upper or lower layer of FbLT constructed by a “sandwich manner,” as shown in Figures 2(a) and 2(b). Then, the confocal images of CD31-immunostained lymphatic vascular network in the artificial tissues were analyzed three-dimensionally by Fiji software; the vessel density was plotted along *Z* axis of the artificial tissues (Figure 2(a)). The FbLT control without hASCs showed the main peak derived from the monolayer of HDLECs (Figure 2(b), green dotted line), and an extra peak was seen in the upper layer (Figure 2(b), control, arrow). The presence of this extra peak is thought to be due to FBS-derived endothelial growth factors in the culture medium [26], which directly faces the tissue surface.

Addition of 25% and 100% of hASCs to the upper layer resulted in an increase of vessel density in this layer depending on the amount of hASCs (Figure 2(b), upper panel). By color labeling of the vessels and analyzing along the depth of the tissue using Fiji software, a high frequency of elongated branches in the upper layer of the tissue was visualized by the addition of 25% hASCs, compared to the control (Figure 2(c)). On the other hand, an addition of hASCs to the lower layer tended to increase the vessel density in this layer (Figure 2(b), lower panel), demonstrating a strong induction of lymphatic vascular formation by hASCs. Quantitative analysis of these results suggested the promoting effect of hASCs on lymphatic vessel network formation in artificial tissue (Figures 2(d)).

To further address the factors in hASCs contributing to lymphatic vascular formation, 55 angiogenesis-related factors in the culture supernatants of tissues were analyzed using Proteome Profiler Human Angiogenesis Array Kit, and a part of their profile is shown in Supporting Information 3. We found that 15 factors were detected at higher levels in ASCLT compared with FbLT, which were also partially increased when 25% hASCs were added into FbLT (Supporting Information 3a). These factors include angiopoietin 1 and 2, endothelin-1, bFGF, FGF-7, HGF, and VEGF-A, which have been reported to promote lymphangiogenesis [27–33]. On the other hand, three factors including amphiregulin, DPPIV, and IGFBP-2 were downregulated in ASCLT compared to FbLT. For VEGF-C, which has been reported to have a specific promoting effect on lymphangiogenesis [34], the results showed low expression levels in both FbLT and ASCLT.

In addition, we investigated changes in the expression of angiogenic factors when HDLECs were added to form lymphatic network in the FbT or ASCT, the artificial tissues consisting only of NHDFs or hASCs, respectively. As shown in Supporting Information 3b, the expression of angiopoietin-2 was dramatically increased in both FbLT and ASCLT compared to FbT and ASCT, respectively. In the presence of HDLECs, the expression ratio of 12 factors in ASCLT was higher than that in FbLT ((ASCLT/ASCT ≧ 1 and (FbLT/FbT) ≦ 1). On the other hand, the expression ratio of three factors including amphiregulin, TMP-4, and VEGF-C in FbLT was higher than that in ASCLT ((FbLT/FbT) ≧ 1 and (ASCLT/ASCT) ≦ 1). These results suggest that the potential of HDLECs in lymphatic vascular formation differs between NHDF- and hASC-based tissues, and hASCs have synergistic effects on more factors than NHDFs.

### 3.3. Subcutaneous Engraftment of ASCLT

**Figure 3:**
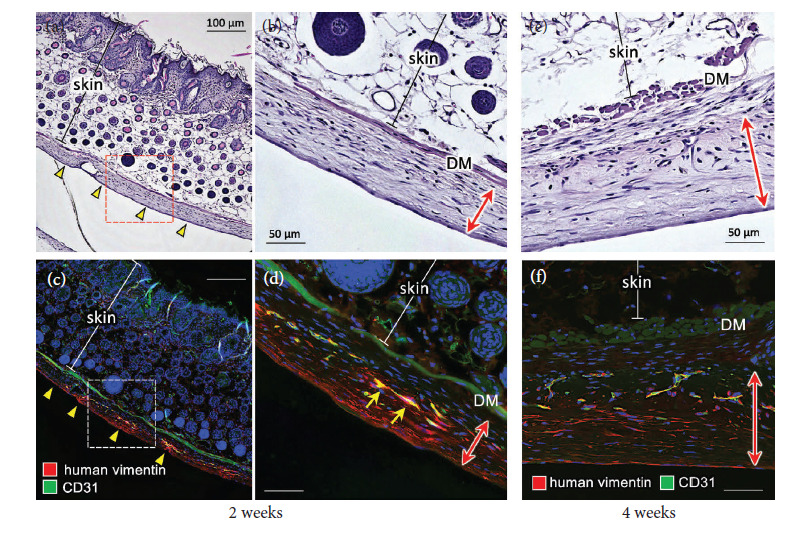
Subcutaneous engraftment of ASCLT to nude mice. The engraftment of ASCLT to gluteal subcutaneous tissue of nude mice was examined. (a, b, e) HE staining; (c, d, f) immunostaining for human vimentin and CD31. (a–d) Two weeks after ASCLT transplantation. The engrafted tissue under the skin is indicated by yellow arrowheads in (a) and (c) or red arrows in (b) and (d) (amplified image from inlet of (a) and (c), respectively). In immunohistochemistry, HDLEC-derived vessels are detected by merged staining of human vimentin and CD31 ((d), yellow arrows). DM, dermal muscle. (e, f) Four weeks after ASCLT transplantation. The engrafted tissue under the skin is indicated by red arrows. DM, dermal muscle. Although the HDLEC-derived vessels remain as shown by merged staining of human vimentin and CD31, the structures are degenerative and the formation of the network or anastomosis with the host lymphatic vessels is not found. The nuclei of the cells in the dark field image are visualized by DAPI (blue color).

The ASCLTs were constructed by the mixture manner as a time-saving method for transplantation experiments. No significant differences in the structure and density of artificial lymphatic vessels were observed between the ASCLTs constructed by sandwich manner and mixture manner (data not shown). Four days after tissue construction, subcutaneous transplantation of ASCLT to the gluteal of nude mice was performed, and then the skin tissues were collected at two and four weeks after transplantation for histological analysis. At two weeks of transplantation, subcutaneous engraftment of the tissues was confirmed by hematoxylin-eosin staining (Figures 3(a) and 3(b), Supporting Information 4a and 4e) and immunostaining for human vimentin (Figures 3(c) and 3(d), Supporting Information 4c and 4g). The thickness of engrafted ASCLTs was higher than that of FbLTs as shown by the arrows in Supporting Information 4a–4c and 4e–4g. Intense Masson-Goldner staining in engrafted ASCLT suggested abundant collagen fibers in fibrotic stromal structure of ASCLT compared to FbLT (Supporting Information 4b and 4f). In addition, TGF-𝛽1 was more abundant in engrafted ASCLT than FbLT (Supporting Information 4c, 4d, 4g, and 4h). The HDLEC-derived vessels in engrafted ASCLT were also observed by overlapped immunostaining for human vimentin and CD31 (Figures 3(c) and 3(d)). In the engrafted tissues, most of the HDLEC-derived vessels did not show an opened lumen (Figure 3(d), yellow arrows). At four weeks of transplantation, the HDLEC-derived vessels in engrafted ASCLT did not develop to be a lymphatic network in the host tissue but showed a degenerated shape in the graft (Figures 3(e) and 3(f)).

### 3.4. Regeneration of Lymphatic Drainage by Gluteal Transplantation of Artificial Lymphatic Vascular Tissues

**Figure 4:**
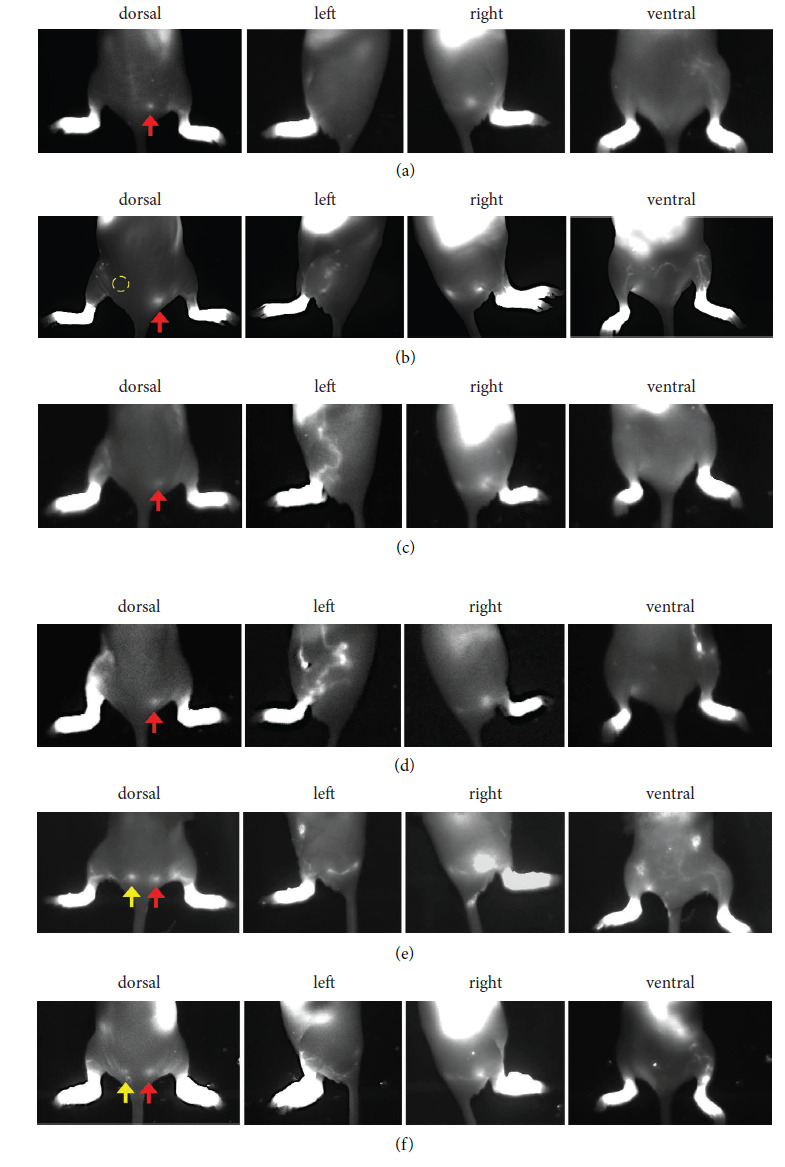
Regeneration of lymphatic drainage by gluteal transplantation of ASCLT visualized by ICG. Subcutaneous transplantation of ASCLT was performed to the gluteal of LDI model mice. After three weeks, 0.5% ICG solution was injected into the foot for visualizing the lymphatic drainage (as shown in Supporting Information 2a). (a) The control without ASCLT transplantation. The drainage in the left limb to gluteal is not detected. (b) The control with subcutaneous transplantation of ASCT (tissue constructed by hASCs alone without HDLECs and lymphatic network) into the gluteal on the route of drainage (yellow circle). The formation of ICG-positive drainage is faintly observed at left side of the limb. (c–f) Four examples of ASCLT subcutaneous transplantation. (c) and (d) show widely spread ICG-positive drainage at the side of the left limb to abdomen. (e) and (f) show the regeneration of the flow to the external sacral lymph node (yellow arrows) suggesting the recovery of intact lymphatic drainage. Red arrow indicates the ICG-positive external sacral lymph node in the intact lymphatic drainage at right limb without lymph node resection.

Subcutaneous transplantation of FbLT and ASCLT into left gluteal (between popliteal and external sacral lymph nodes) of LDI model mice was performed (Supporting Information 2d), and then the regeneration of lymphatic drainage was visualized by injection of ICG. The ICG-positive lymphatic drainage appeared at two weeks after transplantation with FbLT and ASCLT (data not shown). The ICG images at three weeks after transplantation of FbLT are shown in Supporting Information 5. In the control LDI model mice without transplantation, almost no lymphatic drainage was found (Supporting Information 5a). Mice transplanted with non-HDLEC FbT, as a control by two mice, did not show lymphatic drainage (Supporting Information 5b). On the other hand, mice with FbLT transplantation showed the formation of lymphatic drainage from the hindlimb to the side trunk, although drainage to the external sacral lymph nodes was not observed (Supporting Information 5c and 5e, dorsal and left). The routes of drainage in the skin exhibited irregular and diverse shapes (Supporting Information 5c and 5e, left). Similarly, the in vivo images at three weeks after the subcutaneous transplantation of ASCLT are shown in Figure 4. In the control with transplantation of the tissue without HDLECs (ASCT), the drainage of ICG was only faintly detected (Figure 4(b), left). In contrast, mice with ASCLT transplantation showed the formation of ICG-positive lymphatic drainage similar to transplantation of FbLT with irregular and diverse shape of the routes (Figures 4(c) and 4(d), left). Moreover, some cases (2/5) demonstrated the recovery of the drainage to the external sacral lymph nodes in the gluteal corresponding to the original route of drainage (Figures 4(e) and 4(f), dorsal and left).

### 3.5. Regeneration of Lymphatic Drainage by Popliteal Transplantation of Artificial Lymphatic Vascular Tissues

**Figure 5:**
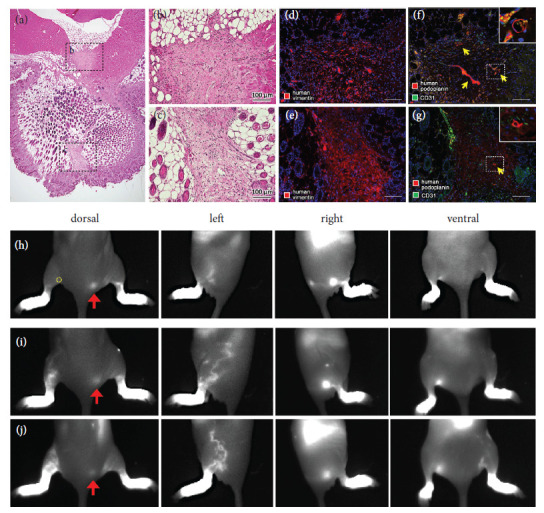
Regeneration of lymphatic drainage by popliteal transplantation of ASCLT. (a–g) Engraftment of ASCLT into deep tissue of left popliteal area. The histological analysis was performed at four weeks after the transplantation to the LDI model mice. (a–c) HE staining. The engrafted tissues are observed in both deep area near the muscle (b) and shallow subcutaneous area (c). (d–g) Immunohistochemistry. (d, e) The serial section corresponding to (b) and (c), respectively. The engrafted tissues are positive for human vimentin. (f, g) The serial section corresponding to (b) and (c), respectively. A few human podoplanin-positive structures are observed (yellow arrows, insets) suggesting that the engraftment of human-derived lymphatic vessels is restricted. The invasion of host vessels with CD31 immunoreaction is observed in the engrafted tissue. The nuclei of the cells in the dark field images were visualized by DAPI (blue color). (h–j) In vivo imaging of ICG-positive lymphatic drainage at three weeks after the transplantation of artificial lymphatic tissues. (h) The control with transplantation of ASCT (tissue constructed by hASCs alone without HDLECs and lymphatic network) into popliteal area (yellow circle). The formation of ICG-positive drainage is faintly observed at left side of the limb. (i, j) Two examples of ASCLT popliteal transplantation. ICG-positive drainage widely spread at the side of the left limb to abdomen. Red arrow indicates the ICG-positive external sacral lymph node in the intact lymphatic drainage at right limb without lymph node resection.

The ASCLTs were also transplanted into the deep tissue of popliteal area where the lymph nodes were resected in LDI model mice (Supporting Information 2e). In the observation of popliteal tissue at two weeks of transplantation, the artificial tissue was engrafted in the deep popliteal part near the skeletal muscle and subcutaneous part (Figures 5(a)–5(e)). These grafts contained human podoplanin-positive vessels suggesting engraftment of HDLEC-derived lymphatic vessels. However, their significant growth and network formation were not observed (Figures 5(f) and 5(g)).

In vivo imaging of LDI model mice at three weeks after popliteal transplantation of ASCT and ASCLT is shown in Figures 5(h)–5(j). As subcutaneous transplantation in Figure 4, popliteal transplantation of ASCLT resulted in the formation of ICG-positive lymphatic drainage with irregular and diverse shape of the routes (Figures 5(i) and 5(j)). The control with transplantation of ASCT (without HDLECs) showed the drainage with faint fluorescence of ICG (Figure 5(h)). Although the popliteal transplantation of FbLT was also examined in two LDI model mice, the formation of ICG-positive lymphatic drainage was not detected (Supporting Information 5f, left).

The results of the transplantation to LDI model mice are also summarized in Supporting Information 6. It should be noted that only 2-3 mice for the controls with negative results were performed. This would be difficult for conclusion. However, all our results, especially those obtained from subcutaneous transplantation of FbLT and ASCLT, suggest that ASC-based lymphatic vascular tissue can induce the regeneration of lymphatic drainage equal to or better than fibroblast-based tissues.

### 3.6. Histological Distribution of Regenerated Lymphatic Drainage

**Figure 6:**
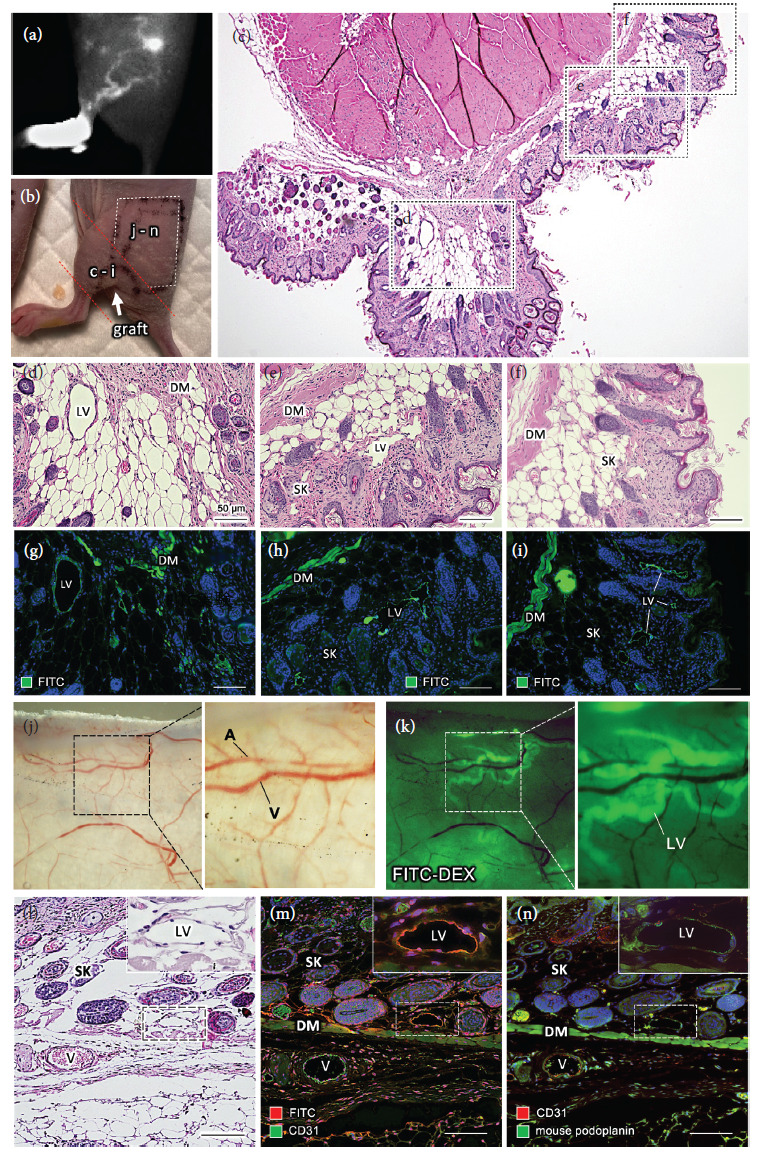
Histological distribution of regenerated lymphatic drainage. (a, b) The side views of LDI model mouse at three weeks after the popliteal transplantation of ASCLT. (a) ICG in vivo imaging. The routes of ICG-positive lymphatic drainage spread in the skin of the left hindlimb to abdomen. (b) Areas of tissue collected after FITC-dextran injection for histological analysis (shown in (c)–(n)). Red dashed line indicates the tissue including popliteal transplantation of ASCLT (graft). White dashed line indicates the area of the cutaneous tissue corresponding to the spread ICG-positive lymphatic drainage. (c) The section of popliteal area with ASCLT transplantation (HE staining). Asterisk (^*∗*^) indicates the graft area. (d-f) High magnification images of (c) (corresponding to (d), (e), and (f) dashed boxes in (c)). (g-i) The corresponding parts of (d-f) in the serial section with immunostaining for FITC. (d, g) Images near the graft area. The lymphatic vessels (LV) in subcutaneous tissue are FITC-positive. Dermal muscle (DM) shows intense nonspecific fluorescence. (e) and (h) and (f) and (i) are images apart from the graft area. FITC-positive lymphatic vessels (LV) were located at subcutaneous tissue (SK) shallower than dermal muscle (DM). (j, k) Fluorescence microscopy of the skin tissue indicated in (b). The subcutaneous side of extracted skin is directly observed. (j) The arteries (A) and veins (V) are recognized by the presence of the blood in bright field. (k) The FITC-positive lymphatic vessels (LV) are visualized by the excitation of fluorescence. (l–n) The serial sections of cutaneous tissue. (l) HE staining. (m) Immunostaining for FITC and CD31. (n) Immunostaining for CD31 and mouse podoplanin. The lymphatic vessels (LV) at subcutaneous tissue shallower than dermal muscle (DM) show FITC and mouse podoplanin-positive (insets of (m) and (n)). V: blood vessels. The nuclei of the cells in the dark field image are visualized by DAPI (blue color).

In most cases of the transplantation with FbLT and ASCLT to LDI mouse model, the formation of ICG-positive lymphatic drainage with irregular and diverse shape of the routes was detected by in vivo imaging. To confirm the distribution of the generated lymphatic drainage in the host tissue, histological analysis was performed at three weeks after popliteal transplantation of ASCLT (Figure 6). After the ICG-positive lymphatic drainage was observed in ASCLT-transplanted LDI mice by in vivo imaging (Figure 6(a)), FITC-dextran was subcutaneously injected to the foot of the transplanted hindlimb. Then, paraffin sections of the popliteal area and gluteal skin, which showed ICG-positive and were predicted to be associated with FITC-dextran loading, were prepared (Figure 6(b)).

In the sections of the popliteal part associated with the engrafted tissue, FITC-positive lymphatic vessels were distributed in the deep subcutaneous layer near the graft (Figures 6(c), 6(d), and 6(g)). In the graft area, FITC-positive human-derived vessels were not found (data not shown). In the area apart from the graft, the FITC-positive vessels were found in subcutaneous tissue and dermis that are shallower than dermal muscles (Figures 6(e), 6(h), 6(f), and 6(i), respectively). These FITC-positive vessels were also immunostained for mouse podoplanin as a mouse lymphatic marker (Supporting Information 7). However, these were not positive for human markers (data not shown).

Next, the gluteal skin was harvested and observed from backside by fluorescence stereomicroscope before paraffin embedding (Figures 6(j) and 6(k)). The fluorescence of FITC was not found in the blood vessels but was found in the meander vessels without blood in the shallow layer of skin (Figure 6(k)). In the skin section, all FITC and mouse podoplanin-positive lymphatic vessels were observed in skin tissue shallower than the dermal muscular layer (Figures 6(l)–6(n)).

These results demonstrated that the ICG-positive and irregular routes of lymphatic drainage are host lymphatic vessels that are developed in the shallow layers of the skin after transplantation of ASCLT.

## 4. Discussion

We have previously reported on the construction of artificial human lymphatic vascular tissue (referred to as FbLT in this article) and its engraftment in mouse fascia and skin [17]. In the present study, hASCs were used for the base of the artificial tissue, resulting in the generation of tubular network in ASCLT by HDLECs. The higher vascular density with sprouting tip formation and more frequent endothelial cell proliferation in the ASCLT, compared to FbLT, suggested a strong promotion of lymphatic vascular formation by the 3D microenvironment of hASC. This activity was also confirmed by the addition of hASCs to fibroblast-based lymphatic vascular tissues. Previous studies have demonstrated the paracrine effects of ASCs on co-cultured lymphatic endothelial cells in their growth, migration, and tubular formation [15, 21, 22]. In the present study, we analyzed the culture supernatants from artificial lymphatic vascular tissues, and the results showed that known lymphatic vascular formation-related factors such as angiopoietin-1, HGF, and VEGF-A increased in a hASC-dependent manner, suggesting that these factors are involved in the promotion of lymphatic network formation by hASCs. As reported in the previous studies, VEGF-A promotes lymphangiogenesis via VEGFR2 signaling [27, 35]. Thus, high concentration of VEGF-A in ASCLT may contribute to the sprouting of the vessels, as VEFGR2 at the tip structure of lymphatic vessels was immunohistologically detected. Furthermore, we previously reported that the angiogenesis-related factors, including VEGF-A and HGF, are increased in the conditioned medium of 3D-cultured hASCs compared with two-dimensional cultures [16]. These conditions could facilitate the promotion of lymphangiogenesis in ASCLT. In our previous study, we also confirmed the promotion of VEGF-A expression in 3D-cultured hASCs and its contribution to vascular formation was suggested [16].

However, VEGF-C, a dominant lymphangiogenesis-promoting factor, was found at very low level, and no increase was detected by the addition of hASCs, suggesting that an involvement of this factor in the lymphatic network formation of ASCLT is low. Previous studies have reported that ASCs express VEGF-C and that VEGF-C expression is further stimulated by exogenous VEGF-C administration [10, 15, 36]. In case of ASCLT, the microenvironment during vascular formation may not contribute to VEGF-C expression. Although the cause of this result is unknown, we expect that early stimulation of hASCs by exogenous VEGF-C may enhance the lymphangiogenic activity in ASCLT.

In histological analysis of ASCLT, the tubular formation was confirmed by light and electron microscopy, demonstrating the formation of lymphatic vessel-like structure as reported in FbLT [17]. However, there was no perivascular differentiation of *α*SMA-positive cells from hASCs or valve formation at the lumen, suggesting that functional lymphatic vessels were not matured in vitro.

In this study, we expected an efficient engraftment and development of artificial lymphatic network in vivo after the transplantation of ASCLT because of the high lymphangiogenic activity of hASC in vitro, as described above. In the transplantation of ASCLT to nude mice, its engraftment in mouse subcutaneous tissue was observed. However, the artificial lymphatic networks tended to degenerate. We have previously reported the transplantation of FbLT on the fascia or in subcutaneous tissue with stable lymphatic vascular engraftment [17]. Therefore, the hASC-related microenvironment in implanted tissue may not allow the stable engraftment of artificial lymphatic vessels. Although the reason for this could not be fully addressed, it can be due to the development of fibrotic tissue upon engraftment of ASC-based artificial tissue, as shown by intense fibrotic staining in ASCLT-derived grafts. In our previous study on engraftment of artificial blood vascular tissue constructed by hASCs and HUVECs, thicker fibrotic graft is found after subcutaneous transplantation compared to the engraftment of the tissue constructed by NHDFs and HUVECs [16]. In this case, interestingly, stable engraftment of human-derived vascular network with differentiation of pericyte- or smooth muscle-like cells from hASCs is found as well as the development of small vessel structures [16]. The formation of fibrotic tissue is known to be promoted by several factors including TGF-*β*1 [37] which was also detected in ASCLT-derived graft. It is also known that TGF-*β*1 directly inhibits lymphangiogenesis [38, 39]. Therefore, the engraftment of HDLEC-derived lymphatic vessels may be suppressed in the hASC-based fibrotic graft, in contrast to HUVEC-derived blood vessels. The matrix stiffness of the tissue also contributes to lymphatic vessel formation. The decreased matrix stiffness enhances in vitro cord-like structure formation of LECs [40]. In the lymphatic development, the decrease of matrix stiffness triggers migration of venous LEC progenitors and lymphatic vessel formation via GATA2 expression [41]. Therefore, inhibition of the fibrosis and decrease of matrix stiffness may improve the engraftment of the artificial lymphatic vessels in ASCLT transplantation.

Even though the subcutaneous engraftment of artificial lymphatic vessels in ASCLT was restricted, the lymphatic drainage was confirmed in mouse LDI model in which the popliteal and ipsilateral subiliac lymph nodes were resected. In our preliminary experiments examining drainage formation by popliteal lymph node resection alone, the intrinsic formation of random drainage appeared within a week, similar to previous reports [42, 43]. However, addition of ipsilateral subiliac lymph node resection resulted in delayed drainage formation at least four weeks postoperatively. This method made it possible to evaluate the effectiveness of artificial tissue transplantation.

Subcutaneous transplantation of ASCLT into LDI model mice resulted in the appearance of ICG-positive and lymphatic drainage at two weeks, whereas the transplantation of control tissues consisting of hASC alone did not. Therefore, the artificial lymphatic vascular network would contribute to the development of new drainage. However, the persistence of engrafted human-derived lymphatic vessels was restricted. Therefore, the efficacy of ASCLT for new drainage might appear in the initial period after transplantation. In the present study, the transplantation of ASCLT in LDI model mice tended to exhibit more effective results compared to FbLT, as shown by the following: (1) the recovery of drainage to external sacral lymph nodes in the gluteal was found in two of five ASCLT-transplanted cases and (2) popliteal transplantation of ASCLT also showed drainage formation. These activities in vivo would reflect the lymphangiogenesis promoted by angiogenic factors that were synergically elevated in the presence of hASCs and HDLECs.

We further analyzed the histological structure of lymphatic tissue in which the drainage appeared after the transplantation of ASCLT. The FITC-positive lymphatic vessels were found in the skin tissue shallower than the dermal muscular layer. Since these lymphatic vessels were negative for human markers, the visualized drainage would be the native vessels forming the superficial lymphatic network that has been described as a part of skin lymphatic structures [1]. Although these can contribute to the reconstruction of lymphatic drainage, the original drainage to the external sacral lymph nodes through the hindlimb and gluteal fascia was not found in most of the FbLT and ASCLT transplantation except for two cases of ASCLT transplantation. Therefore, in the LDI model, anastomosis mediated by artificial lymphatic tissues occurred preferentially within the superficial layer, rather than in the deep fascia (Supporting Information 8). It was considered that the process of anastomosis would occur at the early period after tissue transplantation. At least, ASCLT might provide an appropriate environment for the early regenerative processes by the tissue structures and/or secreted factors. However, as a limitation of this study, the initial anastomosis was not histologically identified. The analysis by in vivo time lapse imaging after transplantation will address this regenerative process. In addition, ASCLT is functionally incomplete due to the lack of valves and collecting vessel-like structures with smooth muscular cells. The structural improvement of artificial lymphatic vessels in vitro may promote the engraftment of functional lymphatic vessels after transplantation.

The results of this study using mouse LDI model suggest the possibility of ASCLT for clinical application of lymphatic regenerative medicine. However, it is necessary to take into account that mice and humans have different skin thicknesses, and humans lack dermal muscles, termed panniculus carnosus muscle, in the trunk and limbs [44, 45]. These suggest that the dynamics of lymphatic drainage between mice and humans may differ significantly. Thus, the therapeutic efficacy on humans using ASCLT is necessary to be carefully evaluated in further studies.

For clinical application of engineered lymphatic vascular tissues, it is also necessary to prepare autologous LECs in a minimally invasive or noninvasive manner. For this purpose, differentiation of LECs from multipotent mesenchymal stromal cells or induced pluripotent stem cells should be established [46, 47]. Endothelial progenitor cells in the blood, called endothelial colony-forming cells (ECFCs), can also be a noninvasive source of LECs, as the presence of lymphatic ECFCs has been reported [48, 49]. In addition, experimental differentiation of LECs from ECFCs is a promising strategy for regenerative medicine of lymphatic drainage by tissue engineering.

## 5. Conclusions

ASCLT showed remarkable artificial lymphatic network formation in vitro. Moreover, its transplantation to the mouse lymphatic drainage regeneration model resulted in the promotion of lymphatic drainage. These results indicate that hASCs can be used as an autologous source for treatment of secondary lymphedema by tissue engineering.

## Data Availability

The image data and analyzed data used to support the findings of this study are available from the corresponding authors upon request

## Supplementary Materials

Supplementary Materials Supporting Information 1: list of antibodies used in this study. Supporting Information 2: schemas for lymph nodes and transplantation of artificial lymphatic vascular tissue to mouse lymphatic drainage regeneration (LDI) model. Supporting Information 3: the profiles of angiogenesis-related factors in the culture supernatants of artificial tissues. Supporting Information 4: comparison of stromal structure between the grafts of FbLT and ASCLT at two weeks after transplantation. Supporting Information 5: regeneration of lymphatic drainage by gluteal and popliteal transplantation of FbLT visualized by ICG. Supporting Information 6: the experiments for ICG-positive lymphatic drainage formation at hindlimb and gluteal of mouse LDI model. Supporting Information 7: promoted lymphatic drainage in cutaneous lymphatic vessels of the mouse with popliteal ASCLT transplantation. Supporting Information 8: the schema of lymphatic drainage regenerated by transplantation of artificial lymphatic vascular tissue in LDI model.
