# Supplementary material for: Lymphatic Drainage-Promoting Effects by Engraftment of Artificial Lymphatic Vascular Tissue Based on Human Adipose Tissue-Derived Mesenchymal Stromal Cells in Mice
Source: J Tissue Eng Regen Med. 2023 Nov 6;2023:7626767. doi: 10.1155/2023/7626767 (PMC11919049; doi:10.1155/2023/7626767)
Supplement: Supplementary Materials — Supporting Information 1: list of antibodies used in this study. Supporting Information 2: schemas for lymph nodes and transplantation of artificial lymphatic vascular tissue to mouse lymphatic drainage regeneration (LDI) model. Supporting Information 3: the profiles of angiogenesis-related factors in the culture supernatants of artificial tissues. Supporting Information 4: comparison of stromal structure between the grafts of FbLT and ASCLT at two weeks after transplantation. Supporting Information 5: regeneration of lymphatic drainage by gluteal and popliteal transplantation of FbLT visualized by ICG. Supporting Information 6: the experiments for ICG-positive lymphatic drainage formation at hindlimb and gluteal of mouse LDI model. Supporting Information 7: promoted lymphatic drainage in cutaneous lymphatic vessels of the mouse with popliteal ASCLT transplantation. Supporting Information 8: the schema of lymphatic drainage regenerated by transplantation of artificial lymphatic vascular tissue in LDI model. [file 7626767.f1.pdf]

Antibody	Company	Cat. No.	Dilution
mouse anti-human vimentin monoclonal antibody	DAKO (Glostrup, Denmark)	M7020	x 100
mouse anti-human podoplanin monoclonal antibody		M3619	x 100
rabbit anti-mouse and human CD31 polyclonal antibody	Abcam (Austin, TX)	ab28364	x 100
mouse anti-alpha smooth muscle actin (αSMA) monoclonal antibody		ab40865	x 200
Alexa Fluor 488-conjugated goat anti-hamster IgG		ab173003	x 200
goat anti-human VEGFR2 antibody	R&D Systems (Minneapolis, MN)	AF357	x 100
goat anti-human VEGFR3 antibody		AF349	x 100
mouse anti-FITC monoclonal antibody	MBL (Nagoya, Japan)	M228-3	x 100
rabbit anti-Prox1 polyclonal antibody	Proteintech (Rosemont, IL)	11067-2-AP	x 100
rabbit anti-phosphorylated histone H3 polyclonal antibody	Santa Cruz Biotechnology, Inc. (Dallas, TX)	sc-8656-R	x 100
Hamster anti-mouse podoplanin polyclonal antibody	AngioBio (San Diego, CA)	11-033	x 100
Alexa Fluor 594-conjugated goat anti-mouse IgG	Invitrogen (Eugene, OR)	A11032	x 200
Alexa Fluor 488-conjugated goat anti-rabbit IgG		A11034	x 200
Alexa Fluor 594-conjugated goat anti-rabbit IgG		A11037	x 200

Supporting information 1

Antibodies used in this study

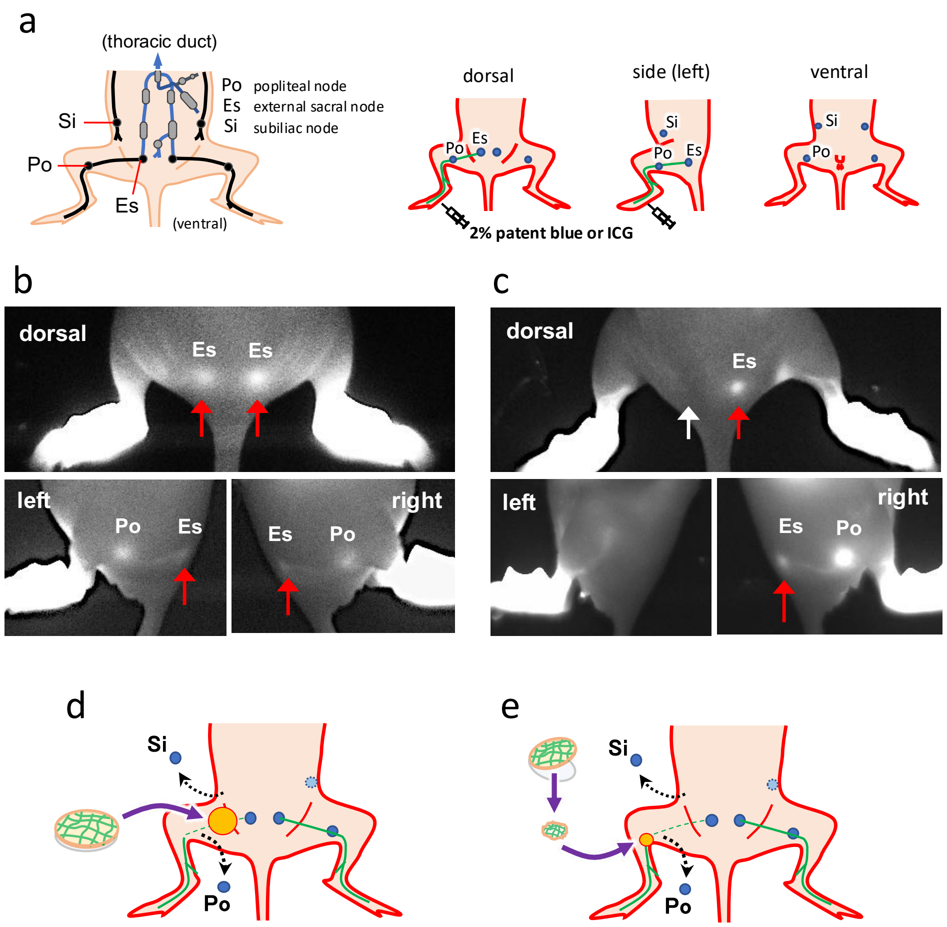

Supporting information 2

Schemas for lymph nodes and transplantation of artificial lymphatic vascular tissue to mouse lymphatic drainage interruption (LDI) model

a: drawing schemas for lymph node and infection site. b: in vivo imaging of lymphatic drainage in intact mouse by indocyanine green (ICG) injection. Subcutaneously injected ICG is drained to popliteal node (PO) and external sacral node (Es). On ICG imaging, Po and Es are visualized as fluorescent dots indicating the intact lymphatic drainage. c: mouse LDI model that had been surgically dissected left Po and subiliac node (Si). At three weeks after the resection, the ICG-positive dots are absent in left Po and Es, showing the disrupted intact lymphatic drainage. Notably, fluorescent dots indicate the intact lymphatic drainage remaining in the right Po and Es. d and e: schemas of subcutaneous transplantation (d) and popliteal transplantation (e) for mouse LDI model.

a

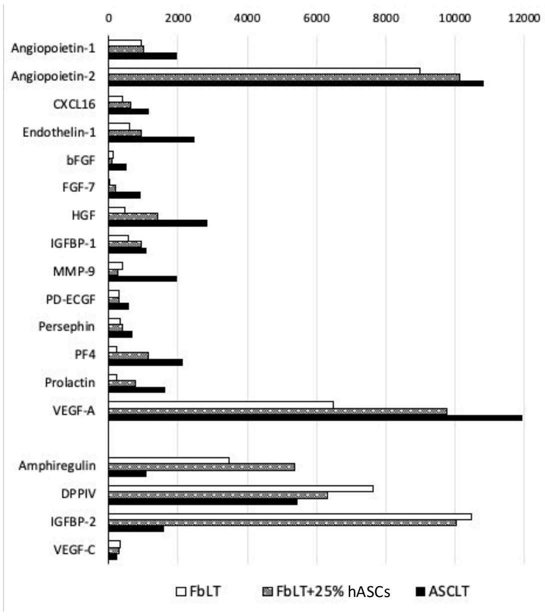

b

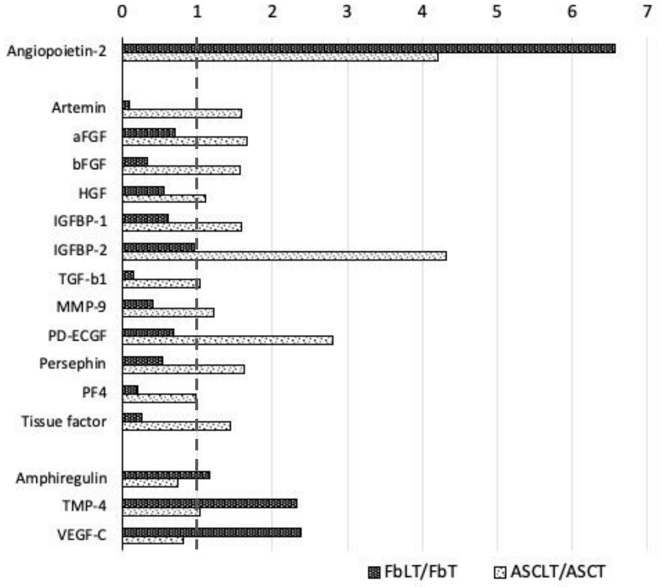

Supporting information 3

The profiles of angiogenesis-related factors in the culture supernatants of artificial tissues

The culture supernatants of artificial tissues were collected, and the angiogenesis-related factors were detected using Proteome Profiler Human Angiogenesis Array Kit. The levels of 55 factors were obtained as immunostained dot blots. a: comparison in FbLT, FbLT + 25% hASCs, and ASCLT. Upper 14 factors tend to increase depending on the amount of hASCs. Lower 4 factors tend to decrease upon the amount of hASCs. b: comparison of the factors in FbLT / FbT or ASCLT / ASCT, for assessing the effect of additive HDLECs to the artificial tissues. The dashed line at score 1 indicates no difference after HDLEC addition.

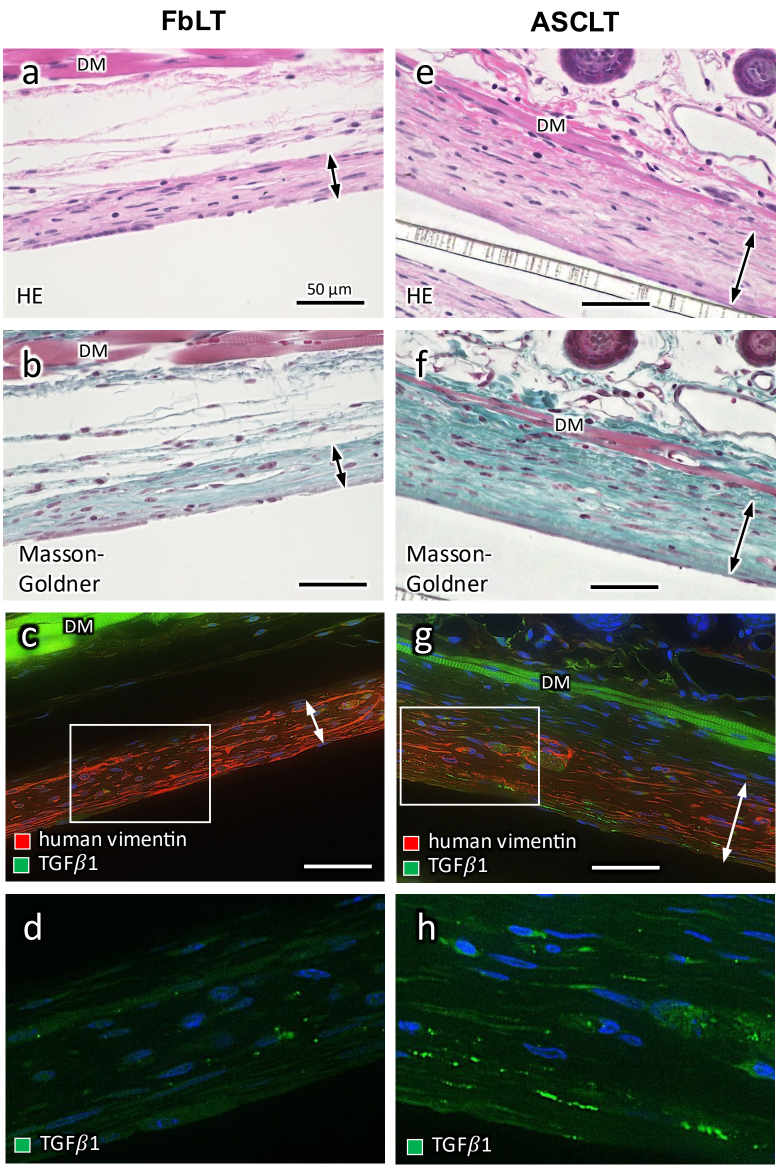

Supporting information 4

Comparison of stromal structure between the grafts of FbLT and ASCLT at two weeks after transplantation

a - d: subcutaneously engrafted FbLT. e - h: subcutaneously engrafted ASCLT. a and e: hematoxylin and eosin staining. The graft of ASCLT shows higher thickness (arrows) and more abundant extracellular structure than that of FbLT. b and f: Masson - Goldner staining. More intense green staining in the graft of ASCLT demonstrates fibrotic stromal structure with abundant collagen fibers. c and g: immunostaining for human vimentin and TGF-β1. Human vimentin-positive area represents the engrafted artificial tissue. d and h: high magnification images of white boxes in c and g, respectively, with immunostaining for TGF-β1. The graft of ASCLT shows more intense signals of TGF-β1 than that of FbLT. DM: dermal muscle. The nuclei of the cells in the dark field image are visualized by DAPI (blue color).

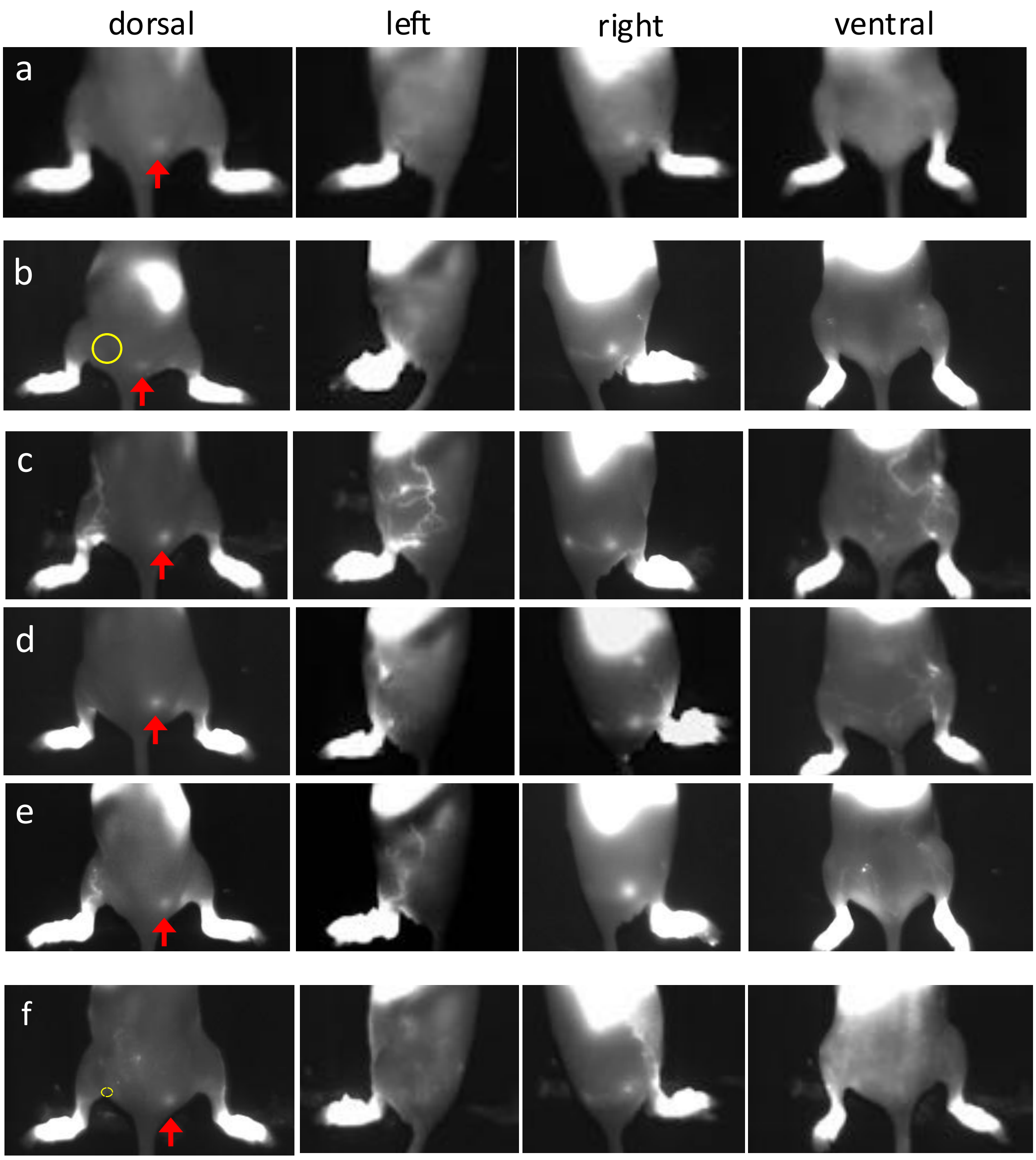

Supporting information 5

Regeneration of lymphatic drainage by gluteal and popliteal transplantation of FbLT visualized by ICG

The FbLTs were subcutaneously transplanted to the gluteal of LDI model mice. After three weeks, 0.5% ICG solution was injected into the foot for visualizing the lymphatic drainage (as shown in supporting information 2 a). a: the control without transplantation. b: the control with subcutaneous transplantation of FbT (tissue constructed by fibroblast alone without HDLECs and lymphatic network) into the gluteal on the route of drainage (b, yellow circle). c - e: three examples of FbLT subcutaneous transplantation. All examples show widely spread ICG-positive drainage from left limb to abdomen. f: an example of FbLT transplantation into popliteal area (yellow circle). No development of ICG-positive drainage is observed. Red arrow indicates the ICG-positive external sacral lymph node in the intact lymphatic drainage at right limb without lymph nodes resection.

artificial lymphatic vascular tissue	subcutaneous transplantation		popliteal transplantation	
control (ND)	–	(n = 3)	–	(n = 2)
control (FbT)	–	(n = 2)	–	(n = 2)
FbLT	+	(n = 5)	–	(n = 2)
control (ASCT)	*Fa*	(n = 3)	*Fa*	(n = 3)
ASCLT	+	(n = 5*)	+	(n = 3)

Supporting information 6
The experiments for ICG-positive lymphatic drainage formation at hindlimb and gluteal of mouse LDI model

The results of the experiments using mouse LDI model are summarized in this table. FbT and ASCT are three-dimensional tissue constructed from NHDFs alone and hASCs alone, respectively (not including HDLECs as well as lymphatic vessels). The lymphatic drainage formation in the left hindlimb to gluteal in LDI model mice are evaluated as follows; (–□: the lymphatic drainage is undetectable. (*Fa*): the lymphatic drainage is faintly detected. (□□: the lymphatic drainage is clearly detected. *: including two cases of flow regeneration to external sacral node.

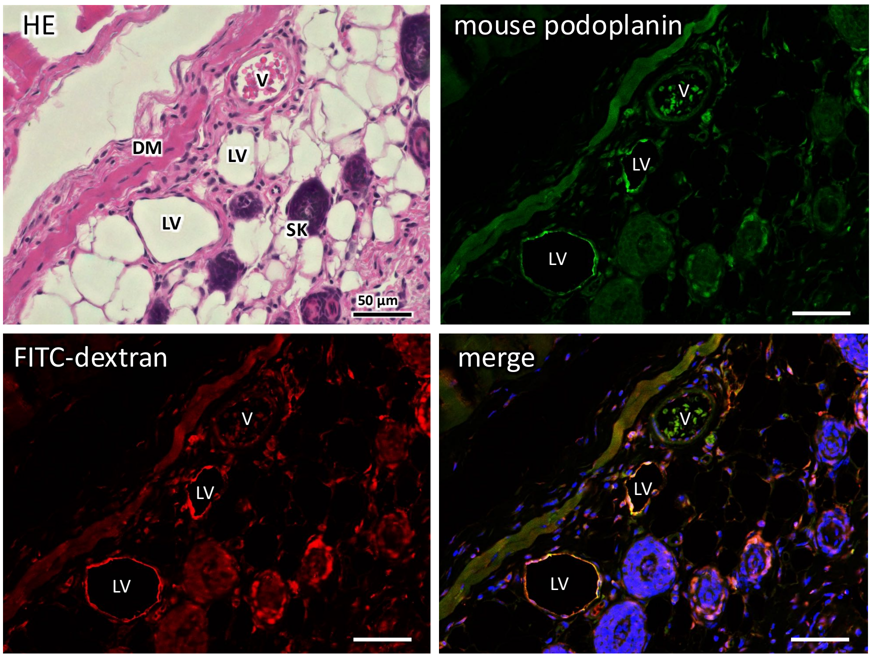

Supporting information 7

Promoted lymphatic drainage in cutaneous lymphatic vessels of the mouse with popliteal ASCLT transplantation

The promoted lymphatic drainage in hindlimb by ASCLT transplantation is visualized by subcutaneous injection of dextran (MW 2,000 kDa)-conjugated FITC. The immunostaining of FITC is observed in the subcutaneous tissue shallower than dermal muscle (DM), consistent with the immunostaining for mouse podoplanin, a marker of the host lymphatic vessels. LV: lymphatic vessels. V: blood vessels. The nuclei of the cells in the merged dark field image are visualized by DAPI (blue color).

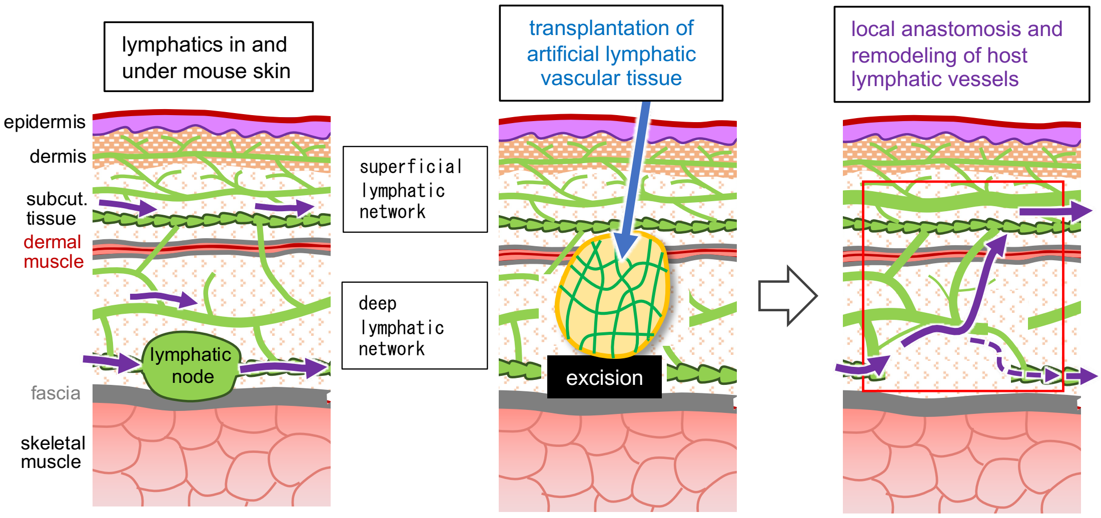

Supporting information 8

The schema of lymphatic drainage regenerated by transplantation of artificial lymphatic vascular tissue in LDI model

Left: popliteal lymph node mainly mediates a deep lymphatic network on the fascia. Middle: the transplantation of artificial 3D lymphatic vascular tissue would result in local anastomoses between existing lymphatic networks, followed by their remodeling to regenerate lymphatic drainage. Right: our results demonstrate that the lymphatic drainage is often regenerated with influx into the superficial lymphatic network (thick purple arrows). However, in some cases of ASCLT transplantation, reconnection to deeper lymphatic vessels is observed (dashed purple arrow).
